# Stimulation of osteoblast activity by homocysteine

**DOI:** 10.1111/j.1582-4934.2008.00104.x

**Published:** 2008-08-11

**Authors:** Markus Herrmann, Natalia Umanskaya, Britt Wildemann, Graziana Colaianni, Thomas Widmann, Alberta Zallone, Wolfgang Herrmann

**Affiliations:** a Department of Clinical Chemistry and Laboratory Medicine, University Hospital of SaarlandHomburg/Saar, Germany; b Center for Musculoskeletal Surgery, Charité-UniversitäsmedizinBerlin, Germany; c Department of Human Anatomy and Histology, University of BariItaly; d Department of Internal Medicine I, University Hospital of SaarlandHomburg/Saar, Germany

**Keywords:** homocysteine, osteoblast, cell culture, alkaline phosphatase, osteocalcin, pro-collage type I N-terminal peptide

## Abstract

Homocysteine (HCY) has recently been linked to fragility fractures. Moreover, HCY activates osteoclasts. Little is known about the effect of HCY on activity of human osteoblasts (OBs). We hypothesized that HCY decreases the activity of OBs. Osteoblasts obtained from tra-becular human bone specimens of eight donors were cultured with conditioned medium. Culture medium was adjusted to 0, 100, 500, 1000 and 2000 μM HCY. After 14 days alkaline phosphatase (AP) activity, pro-collagen type I N-terminal peptide (PINP) and osteocalcin (OC) secretion in the supernatant were measured. After 20 days the formation of mineralized matrix was analyzed. HCY-stimulated AP activity gradually (100 μM HCY: 118%, *P*= 0.006; 500 μM HCY: 125%, *P* < 0.001). At 1000 and 2000 μM HCY the increase of AP activity was reversible (1000 μM HCY: 106%, *P*= 0.317; 2000 μM HCY: 102%, *P* < 0.737). The PINP secretion was also stimulated by HCY reaching a maximum of 260 ± 154 μg/l at 500 μmol/l *versus* 205 ± 94 μ,g/l in controls. After 20 days of culture the formation of bone matrix was increased at 100 and 500 μM HCY. OC secretion was not significantly changed. The results of the present study consistently demonstrate a moderate stimulation of primary human OB activity by increasing concentrations of HCY. However, the magnitude of this effect seems to be less pronounced than recent observations on primary human osteoclasts, suggesting a dysbalance between OBs and osteoclasts in favour of osteoclasts

## Introduction

Osteoporosis represents one of the most common age-related diseases overall [1, 2], affecting about 75 million people in Europe, United States and Japan [1]. Among other common diseases osteoporosis tranks number 1 in women and number 2 (behind chronic obstructive pulmonary disease) in men [2]. Osteoporotic patients are characterized by low bone mass and deterioration of osseous micro-architecture, resulting in decreased bone strength and increased risk of fragility fractures, particularly of the spine, hip and wrist [2–4]. A recent epidemiologic analysis from Switzerland revealed an annual incidence of 491 and 184 osteoporotic fractures per 100,000 persons in women and men, respectively [2]. In the United Staes, more than two million incident fractures occur annually, causing costs of $16.9 billion [5]. By 2025, fractures and costs are projected to grow by 48% to more than three million fractures, incurring $25.3 billion in costs. A comparable situation can be found in Europe and other industrialized countries [6, 7]. Therefore, prevention of osteoporosis by identifying risk factors, or risk indicators, is a major issue.

Previous studies identified advancing age, female gender, early menopause, low body weight, cigarette smoking, alcohol consumption, low calcium intake, a low physical activity level, tallness, prior low-trauma fracture as an adult and history of hip fracture in a first-degree relative, as important risk factors for osteoporosis [8–12]. Recently, an increased plasma homocysteine (HCY) concentration has been suggested to be a new independent risk factor for osteoporotic fractures in elderly persons [13, 14]. The osteoporotic potency of chronic hyperhomocysteinemia (HHCY) could be confirmed by an animal study performed with healthy adult rats [15]. In this study, animals were fed with a 2.4% L-methionine-enriched diet, a 2% homocystine-enriched diet or a control diet for 3 months. Mean plasma HCY was approximately 30 μ,mol/l in the methionine-group, 50 μmol/l in the homocystine-group and <10 μ,mol/l in controls. At the end of the treatment period, the hyperhomocysteinemic animals exhibited a reduction of bone strength (axial compression of the femoral neck until fracture) by up to 40% and a drastic removal of trabecular bone (up to 90%). However, the underlying mechanisms remain obscure. Several studies on human beings observed a correlation between HCY and circulating concentrations of biochemical bone resorption markers, such as desoxypyridino-line cross-links (DPD) [16, 17] and C-terminal telopeptides of collagen type I (ICTP) [18]. First experimental data from our group [19] and Koh *et al.*[20] demonstrate a stimulation of human osteoclasts activity by increasing concentrations of HCY. In addition, extracellular collagen cross-linking, which is important for bone stability might be disturbed [20–22]. However, little is known about the effect of HCY on osteoblast (OB) activity. In hyperhomocysteinemic animals circulating osteocalcin (OC) concentrations have been found to be 40% lower than in controls [15], indicating a reduced OB activity. A reduction of OC expression by HCY could be confirmed in cell culture experiments performed with MC3T3-E1 pre-osteoblastic cells [23]. Another recent cell culture study with human bone marrow stromal cells demonstrated an enhanced apoptosis by HCY stimulation [24]. Contrary to these experimental observations, in human beings HCY positively correlates with the circulating concentration of the OB activity marker OC [16].

Based on existing data, we hypothesized that HCY decreases the activity of primary human OB. Accordingly, we analyzed the influence of increasing HCY concentrations on the activity of primary human OB *in vitro*.

## Materials and methods

### Study design

Trabecular bone specimens, obtained from eight osteoarthritis patients during the implantation of a knee prosthesis, were reduced to small fragments and used to culture primary human OB. The resident OB in the bone samples proliferated and migrated into the culture flask. After proliferation, cells were transferred to multi-well plates (Nunc, Denmark) and cultured with increasing concentrations of HCY. After 14 days of culture, OB activity was quantified by cellular alkaline phosphatase (AP) activity, OC and pro-collagen type I N-terminal peptide (PINP) secretion in the culture medium. Moreover, the synthesis of mineralized bone matrix was analyzed. To guarantee a good reproducibility of the results, each HCY concentration was tested 64 times, 8 donors x 8 repetitions. Informed consent was obtained from all donors and the study protocol was approved by the institutional review board.

### Cell cultures

Freshly isolated trabecular bone samples were cleaned of soft tissue, reduced to small fragments and digested three times in a mixture of 0.7 mg/ml collagenase II (Biochrom Germany) and 2.0 mg/ml collagenase P (Roche Diagnostics, Germany), both from clostridium histolyticum, dissolved in phosphate-buffered saline (PBS; PAA Laboratories, Austria) with gentile agitation for 30 min. at 37°C. After each digestion bone fragments were rinsed 3x with PBS. Then, bone fragments were cultured with α-minimum essential medium (α-MEM), supplemented with 10% fetal calf serum, 100 IU/ml penicillin, and 100μg/ml streptomycin (Invitrogen Germany), at 37°C in a water-saturated atmosphere containing 5% CO_2_. Medium was changed every 3 days. After 3–4 weeks a confluent monolayer of OB had formed. Then, cells were trypsinized and transferred at a density of 10,000 cells/cm^2^ to a 48 multi-well plate (Nunc). In the 48 multi-well plate cells were cultured with the same α-MEM, as described above, that was adjusted to a HCY concentrations of 0, 100, 500, 1000 and 2500 μmol/l, using DL-Homocysteine (Sigma-Aldrich, Germany). Medium was changed every 3 days. After 14 days of culture, OB activity was quantified by the measurement of AP activity, OC and PINP secretion in the supernatant.

### Measurement of alkaline phosphatase activity

AP was quantified by use of the colorimetric ALP assay (Roche Diagnostics) that was adapted to a 48 multi-well plate. After 14 days of culture, medium was removed and cells were washed thoroughly with PBS. Then, 1 ml of p-Nitrophenyl phosphate substrate (99.5 mmol/l, pH 8.5) was added to each well. The substrate was cleaved by the membrane bound AP of the cultured OB forming the yellow dye p-nitrophenol. The p-nitrophenol release is proportional to the AP activity and can be detected photometrically after 10 min. of incubation (linear phase of the reaction) at 405 nm. Each experiment included a positive and a negative control to ensure functionality of the assay.

### Measurement of PINP

PINP secretion in the supernatant was measured with a chemiluminescence immunoassay (Roche Diagnostics) on an Elecsys 2010 automated analyzer (Roche Diagnostics). This sandwich assay uses two monoclonal anti PINP antibodies. The first anti-PINP antibody is bound to biotin and fixes PINP of the sample to streptavidin coated micro particles (solid phase). The second antibody is marked with a Tris(2,2'bipyridyl)ruthenium(II) complex. Magnetic forces bind the micro particles to the surface of an electrode. After washing, the chemiluminescence emission is induced electrically and measured by use of a photomultiplier. Intra- and inter-assay imprecision of this assay are 2.1 and 2.4% at 270 μg/l, and 2.9 and 3.7% at 800 μg/l.

### Measurement of OC

OC, another well-established bone formation marker, was quantified with the N-MID OC assay (Roche Diagnostics) on an Elecsys 2010 automated analyzer (Roche Diagnostics). This sandwich assay uses two monoclonal antibodies against the 1-43 fragment of OC and detects intact OC as well as the stable 1-43 fragment. The first anti-OC antibody is bound to biotin and fixes OC of the sample to streptavidin-coated micro particles (solid phase). The second antibody is marked with a Tris(2,2'bipyridyl)ruthenium(II) complex. Magnetic forces bind the micro particles to the surface of an electrode. After washing, the chemiluminescence emission is induced electrically and measured by use of a photomultiplier. Intra- and inter-assay imprecision of this assay are 4.0 and 6.5% at 15.5 μg/l.

### Mineralization assay

For the mineralization assay OB were transferred at a density of 10,000 cells cm^2^ to a 48 multi-well plate and were cultured for 20 days in α-MEM supplemented with 10% FCS, 50 μg/ml ascorbic acid (Sigma-Aldrich), 10 mM β-glycerolphosphate (Sigma-Aldrich) and 10 nM dexamethasone (Sigma-Aldrich). At the end, mineralized matrix nodules were detected by von Kossa staining. The cells were fixed with cold methanol, washed 3× with water, stained for 10 min. with 5% silver nitrate (AgNo_3_), washed again 3 × with water and finally reduced with sodium carbonate formaldehyde solution (50 g sodium carbonate, 250 ml 40% formaldehyde and 750 ml water) for 2 min. Excessive AgNo3 was removed by incubation with 10% sodium thiosulfate for 5 min. at room temperature. The photomicrographs of mineralized nodules were obtained using a Zeiss Axiovert 40 CFL microscope equipped with an Axiocam MRC digital camera and a personal computer with the Axiovision version 5.4 software package (Zeiss, Guttringen, Germany).

### Statistical analysis

**Fig. 1:**
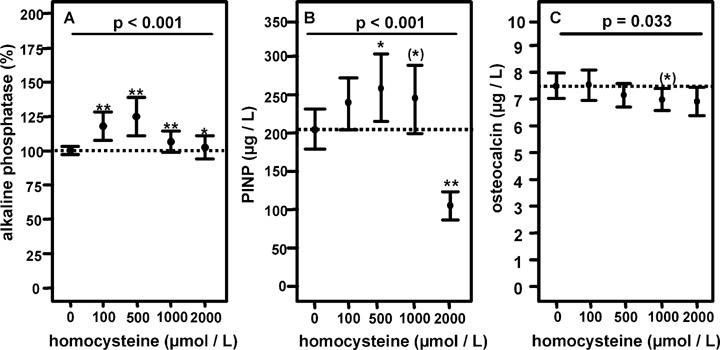
Effect of increasing concentrations of homocysteine (HCY) on primary human osteoblasts. Each condition was tested 64 times (8 donors and 8 repetitions). (**A**) Mean (95% confidence interval) alkaline phosphatase activity. Results are presented as a percentage of the mean AP activity in cultures without HCY (*n*= 8 per individual) as 100%. (**B**) Mean (95% confidence interval) concentration of pro-collagen type I N-terminal peptide in the supernatant. (**C**) Mean (95% confidence interval) concentration of osteocalcin in the supernatant. *P*: *P*-value of one-way ANOVA representing the variation of the group means around the overall mean. (*)*P* < 0.10 (LSD *post hoc test*) versuscontrol cultures without HCY, *P < 0.05 (LSD *post hoc test*) versuscontrol cultures without HCY and **P < 0.001 (LSD *post hoc test*) versuscontrol cultures without HCY.

The descriptive statistics provide data as means ± SD. Means were compared by use of a student's t-test (comparison of two means) or a one-way anova (more than two means) with an LSD (least significant difference) post hoc test. Cultures without addition of HCY were used as controls. AP results from each subject are expressed as a percentage of the control cultures without HCY (mean of 8 wells). The P-values in Fig. 1A–C represent the variation of the group means around the overall mean. The asterisks show the results of the LSD post hoctest. A *P*-value of <0.05 was considered as significant. Calculations were done with the software package SPSS (version 11.0 for windows; SPSS Inc., Chicago, IL, USA).

## Results

### AP activity

AP activity exhibited a noticeable inter-individual variation, which was mainly due to differences in growth behaviour. After 10 min. of substrate incubation, the mean absorption in cultures without HCY ranged between 0.26 and 2.03 optical density (OD). Negative controls exhibited a mean absorption between 0.17 and 0.19 OD. Therefore, we expressed AP activity as a percentage of the mean AP activity in cultures without HCY (*n*= 8 per individual) as 100%. By this method we were able to pool and compare results from all donors (8 donors x 8 repetitions, *n*= 56).

HCY stimulated AP activity gradually, reaching a maximum of 125% at 500 μmol/l (Fig. 1A). At higher HCY concentrations the increase in AP was reversible, which is probably due to toxic effects of HCY.

### PINP and OC secretion

In order to confirm the AP results we measured PINP and OC secretion in the supernatant. The mean PINP concentration in the supernatant of control cultures was 205 ± 94 μg/l. HCY concentrations of 100, 500 and 1000 μmol/l HCY induced a gradual increase of PINP with a maximum of 260 ± 154 μg/l at 500 μmol/l (Fig. 1B). This corresponds to an increase of 27%, which is comparable with the increase of AP.

In cultures without HCY addition mean OC was 7.4 ± 0.8 μg/l, indicating a low variability between donors. In contrast to AP and PINP, the addition of HCY to the culture medium did not change OC secretion (Fig. 1C).

### Formation of mineralized matrix

**Fig. 2:**
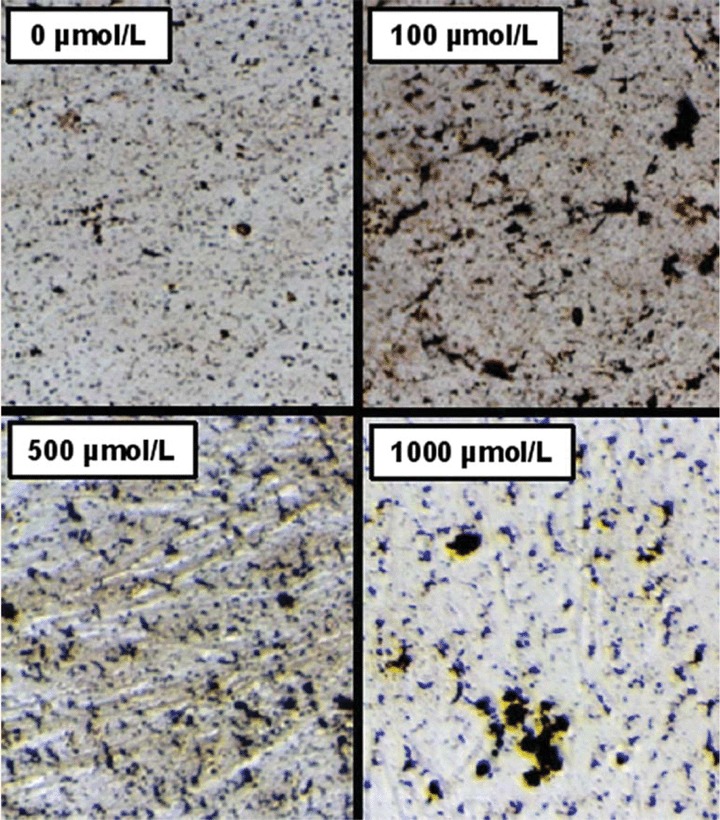
Light photomicrograph (magnification: 400x) of bone nodule formation of primary human osteoblasts cultured for 20 days with increasing concentrations of HCY (von Kossa staining).

As shown in Fig. 2, addition of HCY results in an increased formation of mineralized matrix. This effect was already evident at 100 μmol/l. At higher HCY concentrations this effect was reversible.

## Discussion

The main finding of this study is a moderate stimulation of primary human OB activity by HCY, as indicated by a stimulation of AP activity, PINP secretion and mineralized matrix formation. The maximum increase of OB activity was about 25% and could be observed at a HCY concentration of 500 μmol/l. The strength of our results is due to a high number of repetitions, cells from different donors and the use of well-standardized quantitative measures.

AP, PINP and OC are products of active OB expressed during different phases of OB development. They are considered to reflect different aspects of OB function and of bone formation [25–28]. AP is the characteristic marker for the proliferation phase (days 1–12), PINP characterizes the phase of matrix maturation (days 12–20) and OC is mainly expressed during the matrix mineralization (starting around day 20). Consequently, the OB activity markers used in this study reflect different aspects of OB function. Since the supernatants were sampled at day 14, the missing effects regarding OC can possibly be explained by the later onset of OC expression during the mineralization phase. However, AP and PINP are two well-established markers of bone formation, reflecting OB proliferation and matrix synthesis. Both markers consistently showed a HCY-induced stimulation of OB activity. The results obtained with AP and PINP could be confirmed by the nodule formation assay.

Since experimental studies regarding HCY and bone formation are rare, comparison of our results with other studies is difficult. Our own animal study [15] and a cell culture study performed with MC3T3- E1 preosteoblastic cells [23] demonstrated a reduction of OC expression by HCY. In the animals OC was measured after 12 weeks of HHCY and in the cell culture experiments Sakamoto *et al.* demonstrated a reduction of OC expression in the late phase of culture (up to day 34). In the present study, we collected the supernatant after 14 days of culture, which makes significant changes of OC less probable. Another limitation is that in the animal study and in the cell culture study by Sakamoto *et al.* AP and PINP, the two markers that showed the greatest effects in the present experiments, have not been measured. In addition, Sakamoto *et al.* used MC3T3-E1 preosteoblastic cells. Cell lines are a relatively artificial model and the behaviour of these cells may differ considerably from native OB. The present results were obtained with primary human OB from eight different donors. All test conditions and all cell lines were repeated eight times × 8 donors × 8 repetitions = 64 data points per condition), conferring our results a great statistical strength. Another cell culture study by Kim *et al.* demonstrated an enhanced apoptosis of HS-5 cells (human bone marrow stem cell line) and primary human bone marrow cells. However, this study has several shortcomings. First of all, they analyzed apoptosis, AP and OC expression after 1 and 3 days of culture. This is very early and is not comparable to effects after 14–20 days. At this time point, cells may not have fully recovered from the previous treatment with trypsin. Therefore, the observed effects are questionable. Moreover, AP and OC expression have been quantified by mRNA expression and not by measurement of the protein. Since protein and mRNA expression do not necessarily correspond, the AP and OC data are of limited value. Last but not least, the number of repetitions is not provided in the manuscript, which makes it difficult to interpret the statistic strength of the results. In contrast, the present study demonstrates for the first time a consistent stimulation of primary human OB by increasing concentrations of HCY that is based on a high number of repetitions, cells from many different donors and well-standardized analytic tools.

Compared to circulating HCY concentrations in elderly adults, the HCY concentrations used in this cell culture study were much higher. However, a direct comparison of the concentrations *in vivo* and *in vitro* is difficult. *In vivo*, elevated HCY levels are caused by an impaired degradation of HCY due to B-vitamin deficiencies or an impaired renal function. Low B-vitamin concentrations and impaired renal function are commonly associated with a reduced methylation capacity, which is responsible for many of the adverse effects. *In vitro*, HCY is added exogenously. The B-vitamin concentrations in the culture medium are relatively high and the methyla-tion capacity can be maintained in the presence of elevated extracellular HCY levels. Consequently, *in vivo*, much higher HCY concentrations are needed to induce significant effects.

Osteoporosis is characterized by a dysbalance between OB and osteoclasts [29]. Previous studies by Koh *et al.* and ourselves consistently demonstrated a strong stimulation of osteoclasts by HCY [19, 20]. The maximum increase of tartrate-resistant acid phos-phatase (TRAP) activity was about 50%. In recent cell culture experiments we could confirm a HCY-induced stimulation of TRAP activity by 50%. Moreover, we demonstrated a increase in bone resorption activity up to 400%[30]. The stimulation of OB activity in the present study showed a maximum of 25%, indicating that the HCY-induced stimulation of OB activity is less pronounced than those observed for osteoclasts. Existing data suggests a dys-balance between OB and osteoclasts in favour of osteoclasts as a major pathomechanism for HCY-induced bone loss and reduced bone quality *in vivo*[15].

The main reasons for HHCY in adults are deficiencies of folate, vitamin B_12_ and B_6_ as well as an impaired renal function. Current results from our group showed that B-vitamin deficiencies also stimulate OC activity and lead to elevated HCY concentrations in the supernatant [30]. Bone resorption activity increased up to 200%. This observation is another strong hint for a mainly osteoclast driven deterioration of bone by HHCY. In addition, Kim *et al.* showed a suppression of OB activity in the presence of low vitamin B_12_ concentrations.

Another potential mechanism involved in HHCY-related reduction of bone quality is a disturbed cross-linking of collagen fibrils [21]. In a first study on human beings, Saito *et al.* compared 25 female fracture cases with 25 post-mortem controls and found higher circulating HCY concentrations (∼2 μmol/l) and significantly lower enzymatic cross-links in the bone tissue [31]. However, these results need to be confirmed by others. The potential clinical impact of HHCY for bone health can be deduced from a current large-scale intervention trial by Sato *et al.*[32]. In a population of stroke patients, a group at high risk for fragility fractures, a 2-year HCY lowering treatment by supplementation of folate and vitamin B_12_ resulted in a 75% decrease of hip fractures and overall fractures.

In conclusion, the results of the present study consistently demonstrate a moderate stimulation of primary human OB activity by increasing concentrations of HCY. The results are based on a high number of repetitions, cells from several donors and well-standardized analytic tools. However, the magnitude of this effect seems to be less pronounced than recent observations on primary human osteoclasts, suggesting a dysbalance between OB and osteoclasts in favour of osteoclasts. Future studies need to clarify if the effects obtained by stimulation with HCY can be confirmed by decreasing concentrations of folate, vitamin B_12_ and B_6_.
